# Heart Rate Does Not Reflect the %VO_2__max_ in Recreational Runners during the Marathon

**DOI:** 10.3390/ijerph191912451

**Published:** 2022-09-29

**Authors:** Véronique Billat, Florent Palacin, Luc Poinsard, Johnathan Edwards, Michael Maron

**Affiliations:** 1 Department of STAPS, Université Paris-Saclay, Univ Evry, 91000 Evry-Courcouronnes, France; 2 Laboratory of Neurophysiology and Movement Biomechanics, Université Libre de Bruxelles Neuroscience Institut, 1070 Bruxelles, Belgium; 3 Billatraining SAS, 91840 Soisy-sur-École, France; 4 Department of Integrative Medical Sciences, Northeast Ohio Medical University, Rootstown, OH 44272, USA

**Keywords:** self-pace run, energy cost of running, exercise physiology, endurance, running performance, pacing

## Abstract

Exercise physiologists and coaches prescribe heart rate zones (between 65 and 80% of maximal heart rate, HR_max_) during a marathon because it supposedly represents specific metabolic zones and the percentage of $\dot{V}$O_2max_ below the lactate threshold. The present study tested the hypothesis that the heart rate does not reflect the oxygen uptake of recreational runners during a marathon and that this dissociation would be more pronounced in the lower performers’ group (>4 h). While wearing a portable gas exchange system, ten male endurance runners performed an incremental test on the road to determine $\dot{V}$O_2max_, HR_max_, and anaerobic threshold. Two weeks later, the same subjects ran a marathon with the same device for measuring the gas exchanges and HR continuously. The %HR_max_ remained stable after the 5th km (between 88% and 91%, *p* = 0.27), which was not significantly different from the %HR_max_ at the ventilatory threshold (89 ± 4% vs. 93 ± 6%, *p* = 0.12). However, the %$\dot{V}$O_2max_ and percentage of the speed associated with $\dot{V}$O_2max_ decreased during the marathon (81 ± 5 to 74 ± 5 %$\dot{V}$O_2max_ and 72 ± 9 to 58 ± 14 %v$\dot{V}$O_2max_, *p* < 0.0001). Hence, the ratio between %HR_max_ and %$\dot{V}$O_2max_ increased significantly between the 5th and the 42nd km (from 1.01 to 1.19, *p* = < 0.001). In conclusion, pacing during a marathon according to heart rate zones is not recommended. Rather, learning about the relationship between running sensations during training and racing using RPE is optimal.

## 1. Introduction

Marathon running has increased in popularity since humans first set foot on the moon and with this popularization emerged a new category of runners, namely: “recreational marathon runners” [1,2,3]. These runners are eager [4], less well trained, and experimenters [5,6]. Hal Higdon, an experienced coach, and marathon runner (the first American 1964 Boston marathon finisher; 2:21:55) [7], purports that the key to performance in the marathon is proper pacing. However, ill-advised pacing remains the biggest problem for recreational marathoners as many runners continue to “hit the wall” late in the marathon due to their pacing strategies [8]. More than 80% of runners who “hit the wall” during a marathon report cardio-respiratory distress—increases and decreases in heart rate (HR) and feeling a general malaise and burnout after the 30th km [9]. The marathon is the ultimate exercise in both intensity and duration. It has been shown that elite [10] and recreational runners can reach 100% of their $\dot{V}$O_2max_ during the marathon [11,12]. The ability to sustain a high fraction of $\dot{V}$O_2max_ is a good indicator of marathon performance [5,13,14,15,16]. Indeed, recreational marathon runners often take over twice the time (of the winner) to finish a marathon, and thus sparing muscle glycogen becomes even more critical in order not to suffer from severe fatigue and “hit the wall” [17]. The most widely accepted theory for the association between low muscle glycogen and impaired muscle contractile function is glycogen depletion resulting in a reduction in ATP regeneration. Consequently, the muscles cannot maintain an adequate energy supply to the processes involved in excitation-contraction coupling, leading to an inability to translate muscle drive into an expected force; when this occurs, cramping and fatigue lead to “hitting the wall”. This is supported by observations of decreased phosphocreatine in addition to an increase in free adenosine diphosphate and inositol monophosphate following prolonged glycogen-depleting exercise [18,19].

Runners often pre-plan their pacing efforts using a pacing profile according to past marathon performances [20], feedback from the perceived exertion rate [12], or heart rate and speed. A small pacing error can result in feelings of a subpar performance or severe fatigue and “hitting the wall”. Studies show that previous marathon experience influences a runner’s pace and that there is an interaction between feedback (heart or respiratory rates) and the rate of perceived exertion (RPE) [12,21]. Exercise physiologists and coaches prescribe heart rate zones (between 65 and 80% of maximal heart rate, HR_max_) during a marathon because it supposedly represents specific metabolic zones and the percentage of $\dot{V}$O_2max_ below the lactate threshold, allowing the sparing of muscle glycogen [17]. Heart rate does not serve as an indicator of environmental factors but rather provides an indication of the body’s exercise response when environmental factors change.

A prior study [22] that measured the cardiac output (CO) and the stroke volume (SV), of 14 recreational runners during a marathon which they completed in an average time of 3 h 30 min ± 45 min, showed that they elicited a higher fraction of HR_max_ than the one of their SV and CO (87.0 ± 1.6% vs. 77.2 ± 2.6%, and 68.7 ± 2.8%, for HR, SV and CO, respectively, *p* < 0.05) [23]. Furthermore, data collected during an official marathon race showed that HR was elevated throughout the marathon and increased over time, but without knowing an individual’s baseline $\dot{V}$O_2_ kinetics and HR_max_, there is no sufficient information concerning relative intensity [23].

Indeed, HR is ineffective for estimating the metabolic zone due to cardiovascular drift. A study of 280 recreational marathoners (2 h 30 min–3 h 40 min) showed that the relationship between heart rate increases for each meter run (cardiac cost) [24], and performance speed (m/s) was highly dependent on pacing strategy [25]. A higher increase in cardiac cost was associated with lower performance, resulting in a probable dissociation with the $\dot{V}$O_2_ and HR over time. Consequently, a wrong pacing strategy may lead to an erroneous estimation of an athlete’s metabolic zones, i.e., A non-corresponding increase between HR and $\dot{V}$O_2_. In the same way, performance speed (v, m/s) in running depends on the maximal metabolic power available to the athlete throughout the effort and on the economy of running (1):

(1) $$v=\mathrm{Emax}/C=F\;\times \;\dot{V}O_{2}\max/\mathrm{Cr}$$

where Cr (mlO_2_·m^−1^·kg^−1^) is the energy cost of running the fraction (F, a dimensionless number) is the percentage of $\dot{V}$O_2max_ that can be sustained over a race. Any increase in Cr would inevitably lead to a decrease in v [17]. In this regard, Cr increase with distance completed during simulated competitions that are shorter or identical in duration to the marathon or half marathon. For longer distances, results are equivocal: Cr did not increase after a 6-h ultramarathon, but it increased after 5 h and after 8 h of running at a pace corresponding to 55% and 40% of $\dot{V}$O_2max_, respectively [26]. Despite a drop in running speed that the $\dot{V}$O_2_ level will stay relatively high. We already know that runners will never attain the same percentage of $\dot{V}$O_2max_ as the percentage of HR_max_ and here, the aim of the present study is to test the hypothesis of a possible dissociation between the increases in HR and $\dot{V}$O_2_ of recreational runners during the completion of an actual marathon. Therefore, this disassociation between HR and $\dot{V}$O_2_, could be that most recreational runners will probably be maintaining a marathon pace above their metabolic zone if they use HR as a pacing determinant. This will especially be true in cases where their marathon time are longer than 4 hours. The estimation of the metabolic zone as a percentage of the maximal oxygen uptake (%$\dot{V}$O_2max_) using HR data during the marathon is not reliable due to the different time courses of HR and $\dot{V}$O_2_, which is even more pronounced in longer runs (4 h).

## 2. Materials and Methods

### 2.1. Subjects

Our subjects were ten male, recreational endurance runners (mean ± standard deviation (SD) age: 41.7 ± 7.7 years; weight: 73.2 ± 4.7 kg; and height: 180.5 ± 7.0 cm) (Table 1). 

All study subjects were volunteers and were asked not to modify their habitual training. They were selected for having homogenous physiological and endurance characteristics [27,28,29], and half of the runners had previously run at least one marathon. All subjects declared to have habitually trained 3 to 4 times weekly (50–80 km/week) over more than five years. All subjects performed a high-intensity interval training session once per week of 6 × 1000 m at 90–100% of HR_max_ and a 15–25 km tempo session at 90–100% of their average marathon speed. The study was approved by the Institutional Review Board (IRB Sud-Est V, Grenoble, France; reference: 2018-A01496-49), and all participants were provided with study information and provided written consent.

### 2.2. The Incremental Maximal Test and the Marathon Race

All subjects performed an incremental test (the Université de Montréal track test, Léger and Boucher, 1980) on the road using a portable gas exchange system for determining $\dot{V}$O_2max_, HR_max_, and anaerobic threshold. The UM-TT has been validated as a valid field test of maximal and functional aerobic capacity and suggests that it can be additionally used for exercise prescription [30,31].

The UM-TT was conducted on a 400 m track with cones placed every 20 m. Pre-recorded sound beeps indicated when the subject needed to be near a cone to maintain the imposed speed. A longer sound marked speed increments. The first step was set to 8.5 km·h^−1^, with a subsequent increase of 0.5 km·h^−1^ every minute. When the runner was unable to maintain the imposed pace and thus failed to reach the cone in time for the beep on two consecutive occasions, the test was terminated. The speed corresponding to the last completed step was recorded as the v$\dot{V}$O_2max_ (km·h^−1^). During the UM-TT, $\dot{V}$O_2max_ was confirmed by a visible plateau in $\dot{V}$O_2_ (O_2_ mL·kg^−1^·min^−1^) with a standard increase in exercise intensity, and any indicative secondary criteria (visible signs of exhaustion; HR_max_ ± 10 beats·min^−1^) around the point of volitional exhaustion and an RPE of 19–20.

### 2.3. The Experimental Measurements

The following gas exchange variables were measured: $\dot{V}$O_2_, ventilation (VE), ventilatory equivalents for oxygen (VE/$\dot{V}$O_2_) and carbon dioxide (VE/$\dot{V}$CO_2_). Data from the last 30 seconds of each exercise stage were considered representative measurements of each stage. Maximal $\dot{V}$O_2_ and HR were recorded as the highest values obtained for the last 30 secons period before exhaustion. The Respiratory Compensatory Point (RCP) was identified separately by three researchers as the point where an increase in both VE/$\dot{V}$O_2_ and VE/$\dot{V}$CO_2_ occurred [32]. All plots used in the determinations utilized raw breath-by-breath values. Respiratory gases (oxygen uptake ($\dot{V}$O_2_), ventilation (VE), and the respiratory exchange ratio (RER)) were continuously measured using a telemetric, portable, breath-by-breath sampling system (K4; Cosmed, Rome, Italy). A GPS watch (Garmin, Olathe, KS, USA) paired with the K4 system was used to measure the HR and the speed response (using 5 s data averages) throughout each trial and its validity has been reported [33]. We used the same cardiac belt for the Garmin Forerunner 645 and K4 because it was compatible with both. The subjects self-paced their run without focusing on the cardio-GPS (the display was hidden).

Two weeks later, they completed a marathon wearing the same portable gas exchange system and global positioning system watch (GPS). The incremental test and marathon race were run at the same time (morning), with a 10-day recovery period between the test and the marathon race. The data were collected during France’s 2019 Paris marathon (start times were at 9 a.m.). The temperatures ranged between 10 and 13 °C (between 9 a.m. and 1 p.m.). There was no precipitation, and the humidity averaged 60%. Blood lactate was measured (finger) (Lactate PRO2 LT-1730; Arkray, Japan) just after a warm-up (15 min at an easy pace) and then again three minutes after crossing the finish line.

During the marathon, refreshment points (water, dry and fresh fruit, and sugar) were offered every 5 km and at the finish line, and sponge stations were located every 5 km from km 7.5. At the aid stations, the runners were allowed to remove their masks so that they could drink or eat. To improve comfort, the runners used the mask version with inspiratory valves that reduce inspiratory resistance during high-intensity exercise.

### 2.4. The Variables Used in the Analysis of Results

In accordance with the purpose of this study we analyzed the fractional use of the maximal heart rate and $\dot{V}$O_2max_ and v$\dot{V}$O_2max_. To show the dissociation between these two fractional uses of HR and $\dot{V}$O_2max_ we also analyzed their ratio and compared them at each 5 km section. We also calculated the energy cost of running (Cr in mlO_2_·kg^−1^·m^−1^) i.e., the ratio between the $\dot{V}$O_2_ in mL·kg^−1^·min^−1^ and the speed in m·min^−1^ and the cardiac cost of running in beat·m^−1^ i.e., the ratio between the heart rate (bt·min^−1^) and the speed (m·min^−1^) for every 500 m section.

Given that the runner targets their pace according to a heart rate or speed, we wanted to check that their ratio remained stable by plotting their ratio (%HR/%$\dot{V}$O_2max_ and %HR_max_/%v$\dot{V}$O_2max_). 

### 2.5. Statistical Analysis

All the test variables were reported as the mean ± SD. For each variable, the normality and homogeneity of the data distribution were examined using Shapiro–Wilks, Lilliefors, Anderson–Darling, and Jarque–Bera tests. For analyzing the effect of repetition (within effect) on data average for each 5 km, and the between (group of performance) effect, we applied a repeated measures ANOVA for %$\dot{V}$O_2max_, %HR_max_, %v$\dot{V}$O_2max_ and their ratio (%HR/%$\dot{V}$O_2max_ and %HR_max_/%v$\dot{V}$O_2max_).

We used Pearson’s correlation coefficient to correlate the performance of the % of HR_max_ and $\dot{V}$O_2max_ at each 5 km of the marathon race. We then determined the significance level α = 0.05 for interpreting the statistical tests. Given that we clearly had five runners who achieved the marathon in less than 4 h and five in more than 4 h. Since the slower marathoners (SM) completed their first marathon in more than 4 h, we decided to analyze the influence of the performance level on this dissociation between the heart rate and the oxygen uptake relative to their maximal respective values (%HR_max_ and %$\dot{V}$O_2max_). We therefore checked the normality of distribution before applying the ANOVA for repeated measurement with two factors (repetition for every 5 km and the performance level).

All statistical analyses were performed using XLSTAT software (version 2019.1.1, Addinsoft, Paris, France).

## 3. Results

### 3.1. Maximal Values of $\dot{V}$O_2_ and Heart Rate in the Test UM-TT

Table 2 gives the individual values of $\dot{V}$O_2max_, maximal heart rate and v$\dot{V}$O_2max_ as well as the energy cost of running below the Respiratory Compensatory Point. We can see that the two groups of marathon performance did not have significant differences in these maximal values and in Cr and in their speed at the RCP (in %v$\dot{V}$O_2max_).

### 3.2. VO_2_ and Heart Rate in the Marathon Race

Before examining the marathon data, we must underline that all subjects finished the marathon and that three of them even achieved their personal best (PB) times (Table 1 and Table 2 for comparisons of the PB). The average blood lactate value before that start of the marathon warm-up (1.8 ± 0.8 mM) was significantly lower than the one after the warm-up (2.8 ± 0.7 mM) (*p* < 0.05) (Table 3).

**Figure 1:**
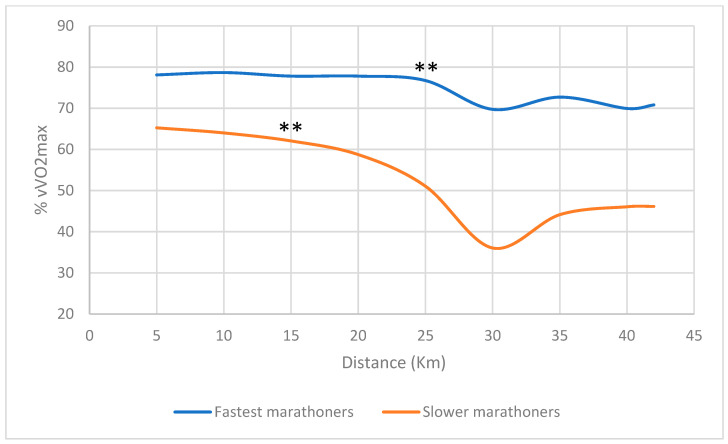
Averages speeds during the marathon of the faster and slower marathoners’ groups expressed in % of v$\dot{V}$O_2max_. For the slower group, the speed was stable until the 15th km and then decreased significantly in between, while for the fastest group, the speed decreased significantly at the 25th km. ** *p* < 0.02).

The slower marathoners (SM) completed their first marathon in more than 4 h (4 h 29–5 h 11 min), while the fastest marathoners (FM) ran it in less than 4 h (2 h 50 min–3 h 45 min). On average, the SM ran at a significantly lower fraction of v$\dot{V}$O_2max_ than the FM group (53.2 ± 3.7 vs. 73.2 ± 5.2 %v$\dot{V}$O_2max_, t = 7.0, *p* < 0.0001) (Figure 1 shows the average value and the one every 5 km). The marathon speed decreased significantly in all the subjects (72 to 59% of v$\dot{V}$O_2max_, *p* < 0.0001) but more in the SM group (65 ± 6 to 46 ± 5 %v$\dot{V}$O_2max_ vs. 78 ± 6 to 71 ± 5 vs. for the FM group, *p* < 0.0001) (Figure 1). For the slower group, the speed was stable until the 15th km and then decreased (*p* = 0.016 between the 15th and the 20th km) while for the fastest group, the speed was stable until the 25th km (*p* = 0.016 between the 25th and the 30th km (Figure 1). The 15th km was reached in the 89th min and the 25th km was reached in the 116th min for the slowest and fastest group, respectively.

**Figure 2:**
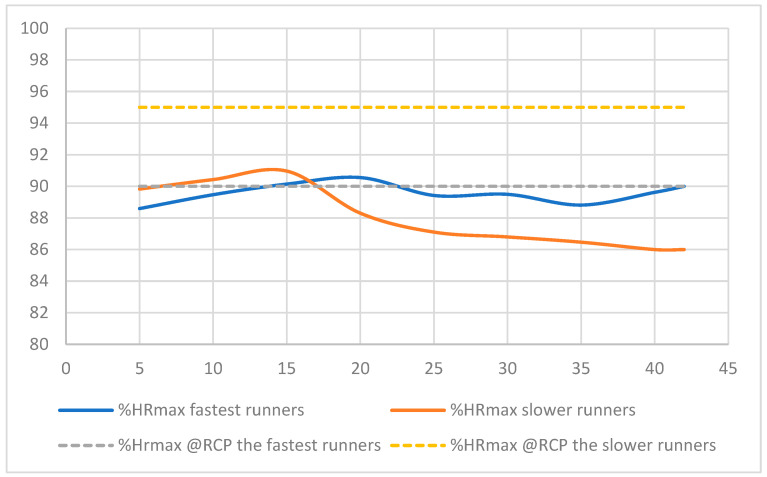
Averages % of HR_max_ during the marathon of the faster and slower marathoners’ groups expressed in % of HR_max_. The % of HR_max_ at the Respiratory Compensatory Point (RCP) was indicated as a reference for each group.

**Figure 3:**
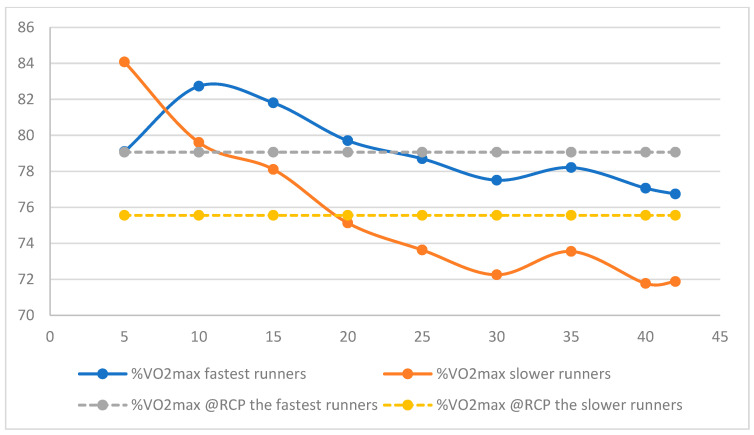
Averages % of $\dot{V}$O_2max_ during the marathon of the faster and slower marathoners’ groups expressed in % of $\dot{V}$O_2max_. The % of $\dot{V}$O_2max_ at the Respiratory Compensatory Point (RCP) was indicated as a reference for each group.

Independent of the groups, the %HR_max_ during the marathon was not significantly different from the %HR_max_ at RCP (89.0 ± 4.4 vs. 93.1 ± 6.6%), and it overlapped in three of the subjects of the FM group (Table 3; Figure 2) [34]. However, in contrast to HR, the average marathon $\dot{V}$O_2_ was significantly lower than the $\dot{V}$O_2_ at the anaerobic threshold (77.3 ±3.6 vs. 84.8 ± 5.1 %$\dot{V}$O_2max_, *p* = 0.001). This relatively high value of %HR_max_ remained stable after the 5th km, remaining between 88 and 91% of the maximal value (*p* = 0.27) (Figure 2), while the %$\dot{V}$O_2max_ decreased from 81 ± 5 to 74 ± 5% (*p* < 0.001) (Figure 3).

**Figure 4:**
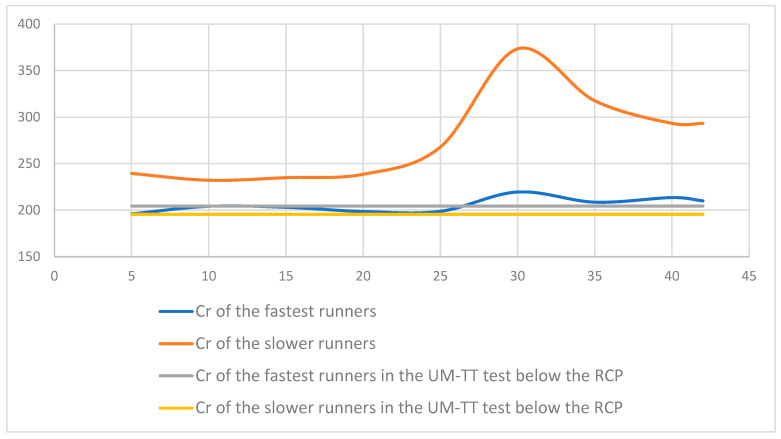
The energy cost of running (Cr) during the marathon of the fastest and slower marathoners’ groups expressed in mL·kg^−1^·km^−1^. The energy cost of running during the below the Respiratory Compensatory Point (RCP) measured in the UM-TT was indicated as a reference for each group.

The energy cost of running in the slower runners during the marathon increased almost significantly at the 30th km (*p* = 0.06) (Figure 4) because some of them started to walk.

## 4. Discussion

Heart rate monitors are utilized primarily to determine the exercise intensity of a training session or race [34]. Compared to other indications of exercise intensity, HR is simple to monitor, is relatively inexpensive, and is used in many situations. However, our results show that HR monitors cannot be used for estimating the energy expenditure or the exercise intensity relative to %$\dot{V}$O_2max_ or v$\dot{V}$O_2max_ during an actual marathon. Indeed, our study showed a dissociation between the fractional use of HR_max_ and $\dot{V}$O_2max_ or v$\dot{V}$O_2max_. Therefore, HR does not allow runners to pace themselves according to a target %$\dot{V}$O_2max_ or even v$\dot{V}$O_2max_ pacing indicator for runners. In our study, the HR remained at a steady state and at a high percentage of HR_max_ (%HR_max_), which was not significantly different from the anaerobic threshold.

Furthermore, the average speed remained far below the speed at the RCP. A prior study [30] measured the cardiorespiratory response for one hour while running at a marathon pace on a treadmill (3 h 40 min 33 min), and it showed that regardless of the marathon finishing time, the runners maintained a marathon pace near the first ventilatory threshold (76.2 ± 6.1% of $\dot{V}$O_2max_), which is well below our second ventilatory threshold value (84.1 ± 5.1 %$\dot{V}$O_2max_) [35].

Our results reveal a dissociation between the %$\dot{V}$O_2max_ and the %HR_max_ due to a speed decrease while the %HR remained stable. This speed decrease was in accordance with our prior study measuring the cardiac output (but not $\dot{V}$O_2_ at that time) during the marathon [22]. Similarly, to our prior study on recreational marathoners [22], we found that HR was 88% HR_max_ vs. 87.0 ± 1.6% in the 2011s study. However, this high value of %HR_max_ does not mean that runner also elicits a high fraction of his stroke volume as reported on our prior study having focused on the cardiac output during the marathon. Indeed, we showed in that cardiac study on the marathon that while the heart rate reached 87.0 ± 1.6% of HR_max_, the % of SV_max_, and maximal cardiac output (CO) which stayed at only 77.2 ± 2.6%, and 68.7 ± 2.8% of their maximal values, respectively [22].

Therefore, according to the present findings and past data, there is clearly a dissociation between the percentage of maximal heart rate and the one of $\dot{V}$O_2max_. However, given that the HR stabilized while the speed decreased, there is an increase in cardiac cost (bt·m^−1^) in accordance with prior marathon race analyses [25].

The ability of runners to sustain the highest fraction of maximal oxygen uptake ($\dot{V}$O_2max_) for long periods has been emphasized as a factor in marathon performance. Indeed, literature accentuates the importance developing high RCP as an %$\dot{V}$O_2max_ so that runners can maintain higher speeds during running without experiencing anaerobic caused fatigue and performance decreases. However, on the marathon, by precocious it is generally recommended that marathon runners stay below the anaerobic threshold (as estimated in this study by the RCP according to the method of Beaver et al. [32]). Our goal was to primarily focus on the ratio between %HR_max_ and %$\dot{V}$O_2max_ during a marathon race. To our knowledge, this has never been reported in the literature and only approximated from laboratory treadmill tests [36] or out on the road [22]. Here we show that it was not possible to estimate with HR a metabolic zone during the marathon race and, more precisely, to associate a given %HR_max_ with a %$\dot{V}$O_2max_ since their ratios increased. Furthermore, this was more pronounced for the ratio between %HR_max_/v$\dot{V}$O_2max_.

Previous reports of the physiological HR-$\dot{V}$O_2_ relationship and race pace characteristics of recreational marathoners were based on treadmill tests using a classical, and unrealistically strict incremental paced protocol [6]. The HR-$\dot{V}$O_2_ relationship is always considered to be linear and constant, which is used to estimate energy expenditure during running conditions [37]. There appears to be consensus in the literature that this method provides a satisfactory estimate of energy expenditure at a group level but is not accurate for individual estimations [38,39]. These studies reported the fractional use of $\dot{V}$O_2max_, HR_max_, using the paradigm of running a marathon at a constant average pace. Significant in our study, we showed that the HR-$\dot{V}$O_2_ relationship does not hold up in actual racing conditions.

Coaches and physiologists continue to define a constant marathon pace as ideal in real-world conditions. However, this paradigm has recently been challenged [25]. A computational study has demonstrated that it was possible to predict the distance at which runners will exhaust their glycogen stores as a function of running intensity [17]. They integrated several physiological variables including the muscle mass distribution, liver, and muscle glycogen densities, and running speed as a fraction of aerobic capacity, i.e., the % of $\dot{V}$O_2max_ [17]. Indeed, the measurement of %$\dot{V}$O_2max_ in actual conditions could improve these predictions. Any increase in Cr would inevitably lead to a decrease in v. Schena et al. [26] showed the effect of a prolonged running trial on the energy cost of running (Cr) in men and women during a 60 km ultramarathon simulation that consisted of three consecutive 20 km laps at a 100 km competition pace. The net increase in serum creatine phosphokinase was linearly related to the percentage increase in Cr observed during the trial. They concluded that, despite increased S-CPK, the running effort was not able to elicit any peripheral or central fatigue or biomechanical adaptation leading to any modification of Cr. They showed that, Cr did not significantly increase after a 60 km trial run at a pace corresponding to best individual performance in the 100 km competition. Therefore, they concluded that human locomotion is a highly stereotyped motor task, and redundant safety factors may operate to preserve a more economical pattern, even in the presence of significant perturbations of a different source. However, in our study, we showed that, once the slowest runner starts to walk at the 30th km, the Cr increased. Indeed, the walk, that is habitually more economical that running [40], is more expensive with fatigue [41]. However, Cr was restored when the runner started to run again at the 35th km mark.

The practical application here is that the degree of metabolic effort varies considerably between individual athletes who run at similar percentages of $\dot{V}$O_2max_. In addition, with higher percentages of a given $\dot{V}$O_2max_, more variability exists between athletes. Learning how to pace oneself by feeling or sensation, which is RPE, combined with running experience may be more advantageous and realistic [21,42]. Variable-paced running has also been demonstrated to be optimal in 280 sub-elite (2 h 30 min–3 h 40 min) marathoners [25]. Indeed, a marathon was recently run in less than 2 h by a man who ran the three fastest marathons ever recorded in a span of three years—Eliud Kipchoge—in the Tokyo Olympic games. We have demonstrated in a prior paper [43] that the best marathons were run according to a pace distribution that is statistically not constant and with negative asymmetry. The utilization of extreme values and oscillations allows for recovery and optimization of the complementary aerobic and anaerobic metabolisms. This suggest new ways to approach the pacing for optimizing endurance performance, and it has recently shown that speed variation can be controlled to maintain a certain RPE in recreational marathoners [12]. Our study showed that it is not accurate to use heart rates to access the aerobic zones for marathon pace, particularly for the slower recreational marathoners. It is essential to highlight that 4 of the 5 runners in the slower group ran their first marathon, in addition to wearing the K4 portable gas-exchange device. Moreover, 3 of 5 runners in the faster marathon group ran their personal best, and all 10 said they were comfortable wearing the K4 device.

Therefore, the goal of self-pacing and staying in a specific zone using HR may be impossible. Heart rates during the marathon were not significantly different from those at the ventilatory threshold determined in an incremental test. The recommendations by many authors for self-pacing using speed or heart rate zones cannot be used for pacing during a competitive event [44,45,46]. We recommend using RPE during the marathon [12], which is clearly a self-paced exercise for which the constant load or speed model cannot be applied [44]. We recommend that the marathoners must maintain their effort at a certain RPE (13–14 on the Borg Scale).

## 5. Conclusions

The most fruitful discoveries are usually made through laboratory and field-based research [47]. By studying recreational runners in an actual marathon race, we discovered that metabolic zones could not be estimated using the heart rate during the marathon. During the marathon, there is a clear dissociation between the observed increase in heart rate and the metabolic responses as $\dot{V}$O_2_ decreases following the decrease in speed. Consequently, the heart rate is unreliable for estimating the metabolic zone (%$\dot{V}$O_2max_) during the marathon. Pacing using heart rate zones during the marathon cannot be recommended, particularly in slower recreational marathoners. These results suggest that the fraction of HR_max_ during the marathon is not stable and increasingly dissociates from F$\dot{V}$O_2max_. We showed that marathon performance over a period between 3 and 5 h did not depend on the factors of performance measured in an incremental test. Rather, it depended more on the running pace early in the first 5th km of the race [12,43]. Big data has shown the consistency of pacing profiles according to performance level. However, it cannot help the individual with race planning, as suggested by the classification of the big data process [20]. The marathon is a special event and open to everyone. It is long and intense and fascinates scientists because the outcome is unpredictable, even for experienced runners. The factors besides exercise intensity which affect exercising heart rates and confound users of HR monitors are the temperature increase during the race (that was not the case since the temperature on that day was maintained between 10 °C at 9 a.m. and 13 °C at 14 h a.m. as recorded in the method section). Furthermore, prior study has shown that the difference between Garmin^®^ and electrocardiography HR values showed that the Garmin Forerunner (we used here) can be used at rest, as well as with walking and running activities of light, moderate, and vigorous intensities such as the marathon race [48].

Pacing during a marathon according to heart rate zones is not recommended. Rather, learning about the relationship between running sensations during training and racing using RPE is optimal.

## 6. Limits of this Study

The limitation of the present study is that we have a small group of runners, but with homogeneous factor of marathon performance according to their $\dot{V}$O_2max_, Cr and RCP in the incremental UM-TT test. However, three of them did not have the marathon experience since it was their first. Furthermore, we neither control the ingesta nor the glycemia in the race and the fact that the slower runners walk at the 30th km could be due to a refueling error during the race. However, it has been demonstrated that the different responses of RPE are explained by the difference in glycogen concentration in muscle, because glucose infusion had no effect on RPE when muscle glycogen content was presumed to be at a normal level and was effective when glycogen in the exercising muscles was presumed to be depleted [49].

## Figures and Tables

**Table 1:** Anthropometric characteristics of the subjects and their personal best in the marathon.

Subjects	Level	Age (years)	Weight (kg)	Height (cm)	BMI
1	High	47	71	175	23.1
2	High	44	82	183	24.5
3	High	33	71	177	22.7
4	High	34	68	181	20.8
5	High	37	74	193	19.9
6	Low	50	71	170	24.6
7	Low	37	66	173	22.1
8	Low	33	77	180	23.8
9	Low	53	75	186	21.7
10	Low	49	77	187	22.0
Mean		41.7	73.2	180.5	22.5
SD		7.7	4.8	7.0	1.5

**Table 2:** The maximal oxygen uptake ($\dot{V}$O_2max_ in mL·kg^−1^·min^−1^); the speed associated with VO_2max_ (v$\dot{V}$O_2max_ (km·h^−1^)); the maximal heart rate HR_max_ (bpm) measured in the UM-TT test.

**Level**	v$\dot{V}$**O_2max_** **(km·h^−1^)**	**HR_max_** **(bpm)**	$\dot{V}$ **O_2max_** **(mL·kg^−1^·min^−1^)**	v@RCP%v$\dot{V}$**O_2max_**	**CR (mL·kg^−1^·km^−1^) %v@RCP**
High	15.9	176	53	96%	214
High	16.5	179	52	92%	204
High	16.8	174	57	86%	203
High	18.5	169	63	87%	212
High	17.0	170	48	91%	189
Low	16.5	183	49	92%	180
Low	16.5	178	53	81%	191
Low	16.0	188	52	91%	217
Low	16.0	175	45	94%	187
Low	16.0	184	53	83%	203
Mean High Level	16.9	174	55	90%	204
SD Group 1	1.0	4	6	4%	10
Mean Level Low	16.2	182	50	89%	196
SD Level Low	0.3	5	4	6%	15
*p* value	0.2	0.06	0.22	0.840	0.333
Mean All the runners	16.6	178	52	89%	200
SD All the runners	0.8	6	5	5%	13

**Table 3:** The marathon time, speeds, oxygen uptake ($\dot{V}$O_2max_ in mL·kg^−1^·min^−1^); the speed associated with $\dot{V}$O_2max_ (v$\dot{V}$O_2max_ (km·h^−1^)) and heart rate in % of the HR_max_ (bpm) measured in the UM-TT test. Vmarathon is the average speed on the marathon; Vmarathon % v$\dot{V}$O_2max_ is the Vmarathon expressed in % of v$\dot{V}$O_2max_; HRmarathon is the average heart rate on the marathon; HRmarathon %HR_max_ is the heart rate in % of HR_max_.

Level	Marathon Times (s)	Vmarathon (km·h^−1^)	Vmarathon %v$\dot{V}$O_2max_	HRmarathon (bpm)	HRmarathon %HR_max_	HRmarathon %HR RCP	Vmarathon %v$\dot{V}$O_2max_	$\dot{V}$O_2_ Marathon %$\dot{V}$O_2max_
High	12694	12.0	75%	169	96%	105%	75%	83%
High	12897	11.8	71%	162	90%	98%	71%	77%
High	12160	12.5	74%	160	92%	110%	74%	78%
High	10200	14.9	80%	148	88%	104%	80%	80%
High	13449	11.3	66%	139	82%	84%	66%	77%
Low	18700	8.1	49%	155	85%	89%	49%	82%
Low	17795	8.5	52%	161	90%	92%	52%	72%
Low	16161	9.4	59%	159	85%	91%	59%	74%
Low	17650	8.6	54%	148	85%	89%	54%	73%
Low	18382	8.3	52%	173	94%	99%	52%	76%
Mean High Level	12280	12.5	74%	156	90%	100%	74%	79%
SD Group 1	1250	1.4	5%	12	5%	10%	5%	3%
Mean Level Low	17737	8.6	53%	159	88%	92%	53%	76%
SD Level Low	979	0.5	4%	9	4%	4%	7%	4%
*p* value	0.008	0.008	0.008	0.999	0.548	0.222	0.841	0.1000
Mean All therunners	15008	10.5	63%	157	89%	96%	63%	77%
SD All the runners	3065	2.3	12%	10	5%	8%	12%	4%

## Data Availability

The data presented in this study are avaible on request from the corresponding author. The data are not publicly avaible due to privacy.
